# Recurrence Rates and Outcomes After First Detected Atrial Fibrillation Episode in a Contemporary Hypertrophic Cardiomyopathy Center

**DOI:** 10.3390/jcdd13100517

**Published:** 2026-10-14

**Authors:** Mauro Rogerio de Barros Wanderley, Martin S. Maron, Jonathan S. Silver, Paul Harnish, Bruce G. Hook, Ethan J. Rowin

**Affiliations:** 1 Internal Medicine Department, Lahey Hospital and Medical Center, Burlington, MA 01803, USA; mdebarr2@lahey.org; 2 Hypertrophic Cardiomyopathy Center, Lahey Hospital and Medical Center, Burlington, MA 01803, USA; 3 Department of Electrophysiology, Lahey Hospital and Medical Center, Burlington, MA 01803, USA

**Keywords:** hypertrophic cardiomyopathy, atrial fibrillation, stroke

## Abstract

Background: Atrial fibrillation (AF) is the most common sustained arrhythmia in hypertrophic cardiomyopathy (HCM), occurring in 20–25% of patients and associated with increased morbidity, stroke risk, and symptom burden. Despite this substantial clinical impact, the understanding of the short-term natural history of AF, including recurrence and the effectiveness of treatment strategies in HCM, remains poorly characterized. Methods: We assessed 2016 patients referred to Lahey Hospital and Medical Center for the diagnosis and subspecialty management of HCM, of whom 341 (17%) had a remote history of AF (≥5 years), and 149 (7%) had AF episodes initially detected between January 2020 and June 2025. These 149 patients were included in this retrospective analysis to allow comprehensive short-term assessment of AF recurrence, treatment interventions, and clinical outcomes from first detected AF utilizing contemporary treatments. Results: AF was first detected at a median age of 63 years (IQR 54–70); 63% were male. Most AF episodes (87%) were symptomatic at presentation. Among 146 patients who converted to sinus rhythm, AF recurred in 80 (54%) at a median of 171 days (IQR 53–278; 29% at 6 months, 43% at 12 months). In patients not undergoing rhythm control after first detected AF episode, 60% had spontaneous recurrence (29% at 6 months, 48% at 1 year), while recurrence rates after antiarrhythmic drug (AAD) initiation were 44% (28% at 6 months, 37.5% at 1 year), and after catheter ablation were 27% (9.8% at 6 months, 18% at 1 year). At last follow-up, 1.9 years (IQR 1.2–2.6 years) from first detected AF episode, 133 of 149 patients (89%) were in sinus rhythm. Seven patients (4.7%) died at a median of 1.16 years (IQR 0.74–1.62) after their first detected AF; estimated survival was 98.6% at 1 year and 96.0% at 3 years. Conclusions: AF recurrence occurs at high rates after the initial detected AF episode in HCM regardless of rhythm-control strategy or AAD use. Catheter ablation was associated with lower recurrence rates. These findings characterize the relapsing natural history of HCM-associated AF and underscore the need for further studies to optimize rhythm-control strategies in this population.

## 1. Introduction

In hypertrophic cardiomyopathy (HCM), atrial fibrillation (AF) is the most common sustained arrhythmia, occurring in 20–25% of patients, and associated with increased morbidity, including stroke risk and symptom burden [1,2]. While there has been much focus and advances on the treatment of heart failure symptoms and sudden death prevention in HCM, there remains a relative paucity of data regarding the natural history of HCM-AF, including recurrence, frequency of episodes, and impact of treatment interventions, particularly within the context of an individual patient analysis immediately after AF is first detected [3]. This is not a trivial point, considering the mechanism of HCM-AF appears unique, driven in part by increased LA pressures from outflow obstruction and diastolic dysfunction, LA dysfunction, increased LA fibrosis, as well as ion-channel abnormalities and genetic determinants [4,5]. Furthermore, treatment interventions for rhythm control, including antiarrhythmic drugs (AAD) and catheter ablation, are less effective in maintaining sinus rhythm in HCM, likely due to these fundamental mechanistic differences [6,7]. To this purpose, we felt it timely to evaluate the timing and frequency of AF recurrence, impact of treatment interventions, and burden of this arrhythmia in a cohort of consecutive HCM patients managed within a tertiary HCM referral center and followed short-term after first detected AF episode.

## 2. Methods

The database of the Hypertrophic Cardiomyopathy Center at Lahey Hospital and Medical Center (Burlington, MA, USA) was assessed with 2016 HCM patients referred to establish diagnosis and subspecialty treatment, including 341 (17%) with remote AF history (diagnosed ≥ 5 years from clinical assessment), while 149 (7%) had an initial AF episode detected between January 2020 and June 2025 and were included for this analysis. After the first detected AF episode, patients were followed for recurrence of AF, AF treatment interventions, and outcomes. Given the retrospective nature of this study, only patients with first detected AF on or after January 2020 were included in order to ensure accurate and comprehensive documentation of short-term AF history within their electronic medical records.

Clinical diagnosis of HCM was based on an echocardiographic or cardiovascular magnetic resonance demonstration of a hypertrophied and nondilated left ventricle (LV) with wall thickness ≥ 15 mm (≥13 mm in setting of family history of HCM or sarcomere gene mutation), in the absence of another cardiac or systemic disease capable of producing a similar magnitude of hypertrophy. Patients with known phenocopies of HCM (e.g., Fabry disease, lysosomal associated membrane protein-2 cardiomyopathy, or amyloidosis) were excluded. Resting LVOT gradient was measured by Doppler echocardiography, and in patients with a resting gradient < 30 mmHg, provocable obstruction was assessed with the Valsalva maneuver and/or exercise echocardiography and defined as a peak gradient ≥ 30 mmHg. 

Patients were diagnosed with AF based on documented episodes on any form of cardiac monitoring and confirmed by treating cardiologists, including electrocardiograms, Holter monitors, wearable digital devices, or device interrogations from pacemakers or ICDs. Because of the retrospective design, the index event was the first clinically documented AF episode, referred to here as first detected AF in keeping with contemporary nomenclature [8]. As part of clinical evaluation in our center, patients without AF history and without cardiac implantable devices underwent ambulatory rhythm monitoring for 1 to 2 weeks on a yearly basis, in addition to electrocardiograms at clinic visits and symptom-triggered monitoring.

Paroxysmal AF was defined as an episode that self-terminated within <48 h. Persistent AF was defined as an episode that lasted >48 h or required chemical or electrical cardioversion. Permanent AF was defined as continuous AF in patients for whom no further attempts of rhythm control were planned, either due to paucity of AF related symptoms and/or after failure of previous attempts of rhythm control. Chemical cardioversion was defined as restoration of sinus rhythm after administration of AAD, while electrical cardioversion was restoration of sinus rhythm following direct current cardioversion. Patients not undergoing a rhythm control strategy (either AAD or undergoing AF ablation) for treatment of first detected AF were followed for spontaneous AF recurrence (i.e., recurrence rate in the absence of rhythm control interventions), while patients were also followed for AF recurrence after initial attempts for rhythm control with either AAD administration or after AF ablation. Patients treated with AAD who had AF recurrence and subsequent ablation were included in the AAD group and subsequently crossed over into the AF ablation subgroup. As a result, patient follow-up periods were not mutually exclusive between groups: 27 patients crossed over from spontaneous recurrence into the ablation group, and 8 patients from the AAD into the ablation group. After interventional treatment with catheter ablation therapy or Maze surgery, a 90-day blanking period was established [9].

European Heart Rhythm Association (EHRA) symptom classification for atrial fibrillation, a semi-quantitative scale that grades severity of AF-related symptoms based on impact on daily activities, was quantified at the time of first detected AF, with class 1 reflective of no symptoms, class 2a of mild symptoms, 2b of moderate symptoms, 3 of severe symptoms, and 4 of disabling symptoms as previously described [10].

All statistical analyses were performed using Stata version 19 (StataCorp LLC, College Station, TX, USA). Continuous variables were assessed for normality using the Shapiro–Wilk test and are presented as mean ± standard deviation (SD) if normally distributed, or median (interquartile range [IQR], 25th–75th percentile) if non-normally distributed. Categorical variables are expressed as counts and percentages. Freedom from AF recurrence during follow-up was estimated using the Kaplan–Meier method, and differences between groups were compared using the log-rank test, with follow-up initiated after first detected AF, at AAD initiation, or after a 90-day blanking period from the ablation procedure and ended at first AF recurrence, crossover to another strategy, death, or last follow-up. To account for the blanking period in patients undergoing AF ablation resulting in a comparatively shorter follow-up period, analysis of AF recurrence was made for 12 months in patients followed for spontaneous recurrence or those initiated on AAD, as compared to after 15 months for those undergoing ablation.

Associations between clinical and imaging variables and AF recurrence were evaluated using univariate Cox proportional hazards regression analysis. Univariate analysis for AF recurrence was performed in the 98 patients who did not initially receive rhythm control. A multivariable Cox proportional hazards model was fitted in these 98 patients using covariates selected a priori from the literature, rather than from univariate *p*-values, to limit overfitting, including age, LA transverse dimension, resting LVOT obstruction (gradient ≥ 30 mmHg; yes/no), sex, BMI, and NYHA class ≥ II (yes/no), and the proportional hazards assumption was assessed using Schoenfeld residuals. Hazard ratios (HRs) with 95% confidence intervals (CIs) were reported. A two-sided *p*-value < 0.05 was considered statistically significant.

## 3. Results

AF was first detected in 149 patients at a median age of 63 years (IQR 54–70), including nine patients (6%) younger than 30 years and 43 patients (29%) between 30 and 50 years; 63% were male. Patients were followed for a median of 1.9 years (IQR, 1.2–2.6 years) from first detected AF.

HCM was initially diagnosed a median of 2.9 years (IQR 0.03–10.6) before AF, including 48 patients (32%) in whom AF led to the initial identification of HCM. Maximum wall thickness was 19 ± 3.9 mm, including 57 patients (39%) with resting left ventricular outflow tract (LVOT) obstruction (gradient ≥ 30 mmHg) and an additional 22 patients (15% of the cohort) with provocable obstruction (≥30 mmHg after Valsalva or exercise), while 4 (2.7%) had end-stage HCM with LV systolic dysfunction (EF < 50%). LA transverse dimension was 46 ± 7 mm, and HCM-AF score at time of first detected AF was 23 ± 4, with the majority having an HCM-AF score of ≥22 consistent with high risk for AF development (n = 101; 70%) [11].

Prior to first detected AF, 65 (44%) patients were asymptomatic (NYHA class I), 54 (36%) class II, and 30 (20%) had more advanced symptoms (class III). Notably, comorbidities including hypertension (55%), obstructive sleep apnea (28%), and obesity (BMI ≥ 30 kg/m^2^; 46%) were common (Table 1).

First detected AF. The first detected episode was paroxysmal in 98 patients (66%) and persistent in 51 (34%). Most patients (n = 130; 87%) were symptomatic during atrial fibrillation episodes, including EHRA class 2a in 40 (27%), class 2b in 34 (23%), class 3 in 25 (17%), and class 4 in 31 (20%), while 19 patients (13%) were class 1/asymptomatic. In these 19 patients, asymptomatic AF was detected by routine annual ambulatory monitor in six patients, by device interrogation in 5, and on routine ECG during outpatient evaluation in 8.

At first detected AF, 87 patients (58%) self-converted, approximately half of them after additional beta-blocker or calcium channel blocker (BB/CCB) therapy (n = 43, 29%), including 14 BB/CCB-naive patients, and 29 after dose uptitration. Twelve patients (8%) underwent pharmacologic cardioversion with an antiarrhythmic drug (amiodarone in 10 and sotalol or dronedarone in 1 each), and 44 (30%) underwent electrical cardioversion. Three patients (2%) underwent catheter ablation as an initial rhythm-control strategy because of severe symptoms, whereas 3 (2%) elected to remain in permanent AF with a rate-control strategy alone due to minimal symptoms.

AF recurrence. In the 146 patients with restoration of sinus rhythm, AF recurred in 80 (54%) of patients, at a median time to recurrence of 171 days (IQR, 53–278), with a rate of recurrence of 29% at 6 months and 43% at 12 months. AF recurred within 12 months in a similar proportion of patients who presented with paroxysmal and persistent AF (44.9% vs. 40.8%, log-rank *p* = 0.52). In the 80 patients with recurrent AF episodes, 43 (29%) had two total episodes, 29 (20%) had three or four episodes, and 8 (5%) had five or more episodes over a median follow-up of 690 days (IQR, 433–966).

**Figure 1:**
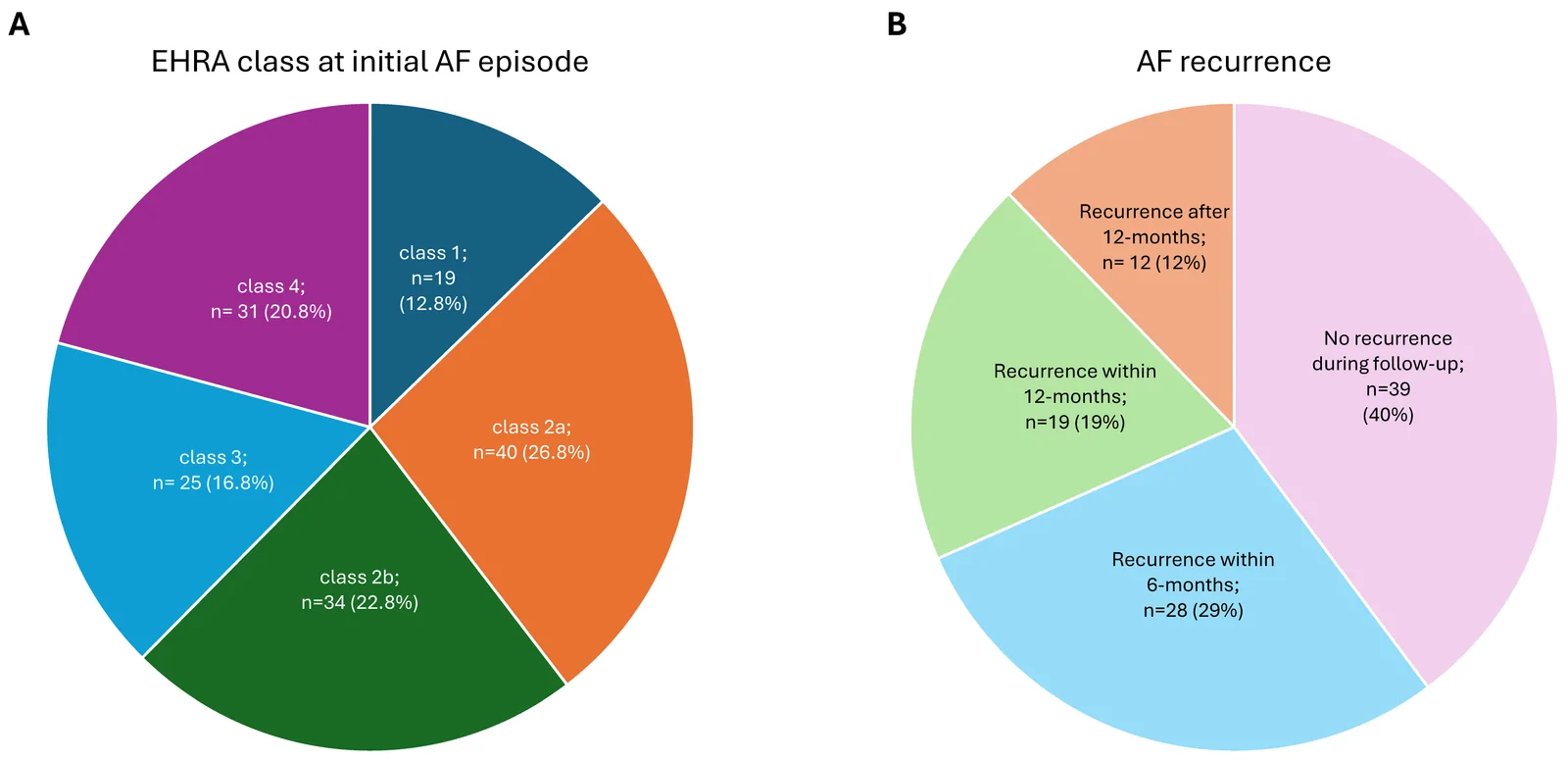
Symptom status and atrial fibrillation recurrence in HCM. (**A**). EHRA classification of symptoms in 149 HCM patients with AF, associated with some symptoms in 87% of patients. (**B**). Freedom from AF recurrence in 98 patients not undergoing rhythm-control treatment with catheter ablation or antiarrhythmic drugs after first detected AF.

When restricted to the 98 patients who did not initially receive a rhythm-control strategy, 59 (60%) experienced spontaneous recurrence, with a median observed time to recurrence of 198 days (IQR, 62–309); AF recurrence of 29% at 6 months and 48% at 1 year (Figure 1). In univariate and multivariable analyses, no individual variable was significantly associated with AF recurrence (Table 2).

A total of 32 patients were initiated on an AAD as their initial rhythm-control strategy. Of these, 7 were started on sotalol, 20 on amiodarone, 1 on dofetilide, and 4 on disopyramide. After AAD initiation, AF recurred in 14 of these 32 patients (44%); AF recurrence was 28% at 6 months and 37.5% at 1 year.

Forty-nine patients underwent ablation at a median of 225 days (IQR, 141–366) after their first detected AF, including 8 (16%) who underwent the procedure following AAD failure. Over a median follow-up of 525 days (IQR, 327–721) after ablation, AF recurrence occurred in 13 patients (27%); 9.8% at 6 months and 18% at 1 year. Of these 13 patients, 7 underwent repeat ablation, 2 were started on AAD therapy (amiodarone and dofetilide), while the remaining 4 opted not to change therapy. In the 49 patients undergoing ablation, 40% underwent pulsed field ablation (PFA), 31% cryoablation, and 29% radiofrequency ablation (RFA). AF recurrence was not significantly different in patients undergoing PFA compared to thermal ablation (RFA or cryoablation) (3/20 [15.0%] vs. 10/29 [34.5%], respectively, log-rank *p* = 0.56). Among 21 patients who underwent AF ablation as an initial rhythm-control strategy (i.e., without prior attempt at AAD), only 4 (19%) experienced AF recurrence during follow-up; the recurrence rate was 9.5% at 1 year (Table 3). Baseline characteristics of patients treated with AAD (n = 32) or catheter ablation (n = 49) are presented in Supplementary Table S1.

**Figure 2:**
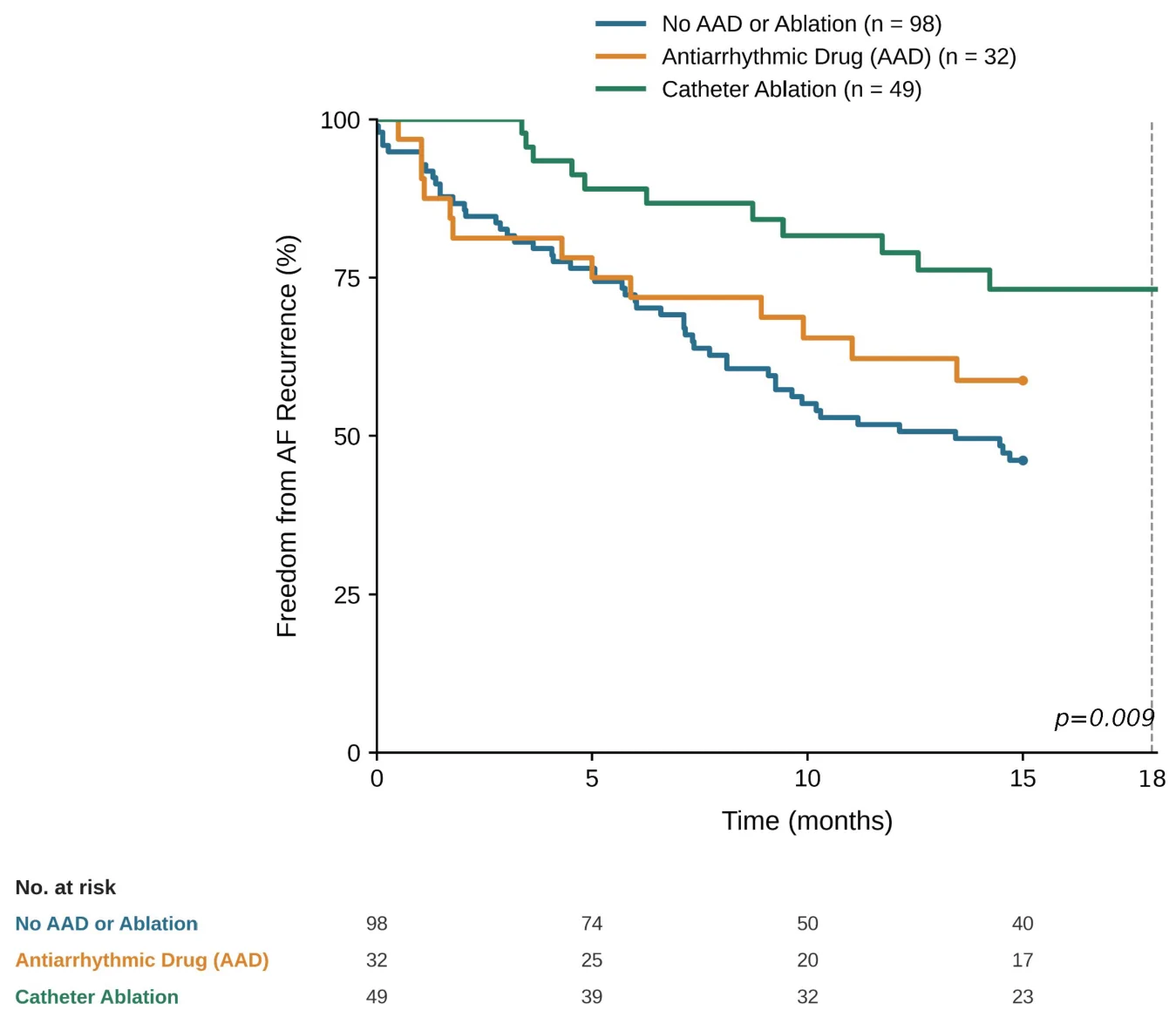
Recurrence of atrial fibrillation after treatment interventions in HCM. Kaplan–Meier curve showing AF recurrence in patients treated with antiarrhythmic drugs, catheter ablation, and those followed for spontaneous recurrence at 1 year after the blanking period. Patients were followed for spontaneous recurrence and antiarrhythmic drugs for up to 12 months, whereas those undergoing ablation were followed for up to 15 months to account for a 3-month blanking period post-ablation.

Recurrence rates of AF were lowest in the 49 patients undergoing ablation compared to the 32 patients initiated on AAD or the 98 patients treated solely with rate control at 1 year after blanking period (12 months from first detected AF or AAD initiation, and 15 months from ablation accounting for 3-month blanking period; *p* = 0.009, Figure 2).

Asymptomatic AF. First detected AF was asymptomatic in 19 patients and diagnosed incidentally during interrogation of an implanted device in 5, through ambulatory monitoring in 6, and on routine ECG at the time of an outpatient clinical visit in 8. Overall, 9 (47%) of these initially asymptomatic patients experienced recurrence during follow-up; 21% at 6 months and 42% at 1 year. Among these 19 patients, 4 (21%) ultimately developed symptomatic AF, most commonly presenting with palpitations, and required rhythm control with catheter ablation (n = 3) or AAD therapy followed by ablation (n = 1).

Stroke and anticoagulation. An ischemic stroke that was presumed cardioembolic occurred in 11 patients (7%) preceding AF detection. AF was subsequently identified during the immediate work-up of the stroke in three patients, and in the remaining 8, a median of 0.38 years after the stroke event. Each of these 11 patients was initiated on anticoagulation once AF was identified.

**Figure 3:**
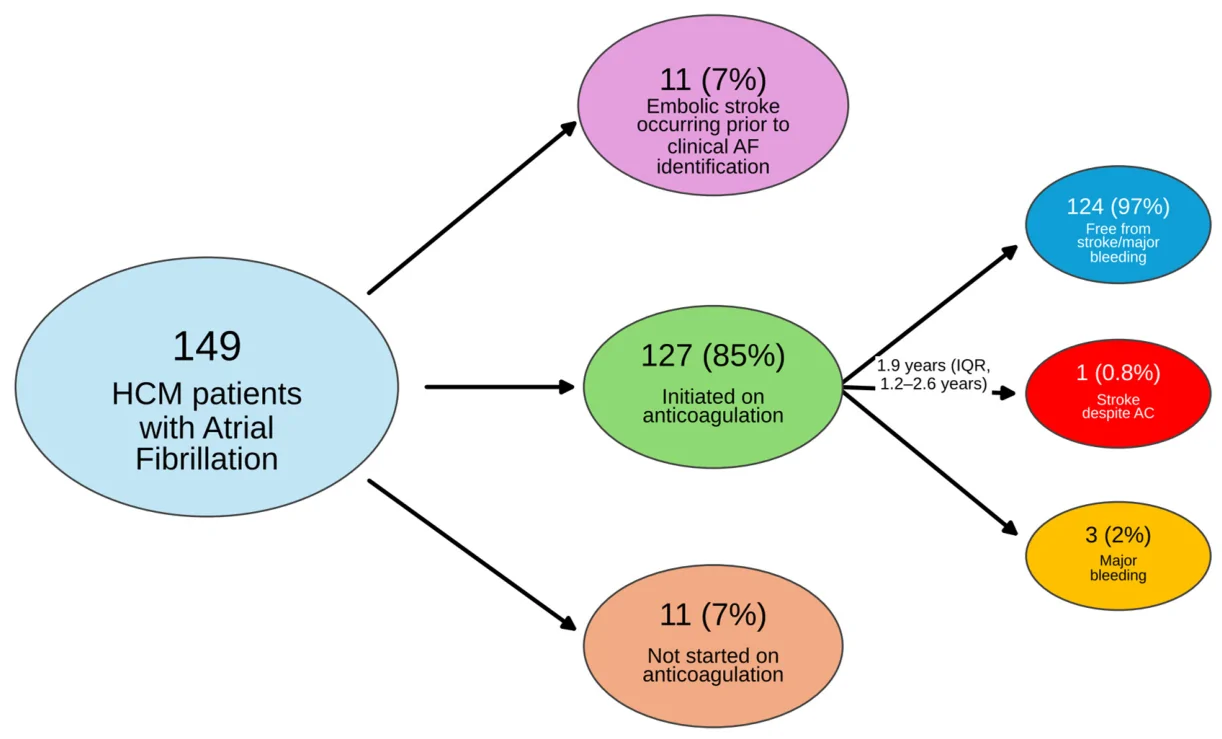
Anticoagulation use, major bleeding and stroke in HCM. Of 149 patients with HCM and AF, an ischemic stroke occurred before first detected AF in 11 (7%), while anticoagulation for stroke prevention was initiated in 127 patients at the time of AF identification, and was not started in 11 patients. In the 127 patients initiated on anticoagulation, only 1 (0.8%) had an embolic stroke, while 3 (2%) had major bleeding events (including one with fatal hemorrhagic stroke). AC, anticoagulation.

In the remaining 138 patients, anticoagulation for stroke prevention was initiated at the time of AF identification in 127 patients and was not initiated in 11 patients, 2 because of prior major bleeding risk and 9 who declined. Among patients receiving anticoagulation, only 1 experienced an embolic stroke (0.8%), occurring 9 days after electrical cardioversion. In contrast, major bleeding events occurred in three patients on anticoagulation (2%), including intracranial hemorrhage in 1 and gastrointestinal bleeding in 2 (Figure 3).

AF outcomes. At last follow-up, 142 of 149 patients survived, including 133 (89%) in sinus rhythm, while 7 (5%) were in persistent atrial fibrillation (with plans for ongoing AF treatment), and 2 were in permanent atrial fibrillation. At last follow-up, 135 patients (91%) had no or mild symptoms (NYHA class I in 60%, class II in 30%), while the remaining 14 (9%) had NYHA class III symptoms.

Seven patients died at a median age of 68 years (IQR, 60–77), with a median of 1.16 years (IQR, 0.74–1.62) after first detected AF. Estimated survival was 98.6% at 1 year and 96.0% at 3 years. Of the seven patients, 3 died from HCM-related causes: complications post-surgical myectomy, advanced conduction disease after declining pacemaker, and intracranial hemorrhage on anticoagulation (one each), while the remaining 4 died from non-HCM-related causes (Table 4).

Non-HCM deaths: one related to complications of alcohol abuse, two due to multiple non-cardiac comorbidities, and one death was secondary to sepsis. NYHA class and sinus rhythm at last follow-up are expressed as a percentage of all 149 patients.

## 4. Discussion

In patients with HCM, AF is the most common sustained arrhythmia and is associated with significant morbidity, placing patients at risk for stroke and often resulting in worsening symptom burden [12]. However, despite the impact of this arrhythmia on outcomes in HCM, AF remains under-studied as compared to other adverse disease pathways in HCM such as sudden death risk and progressive HF related to outflow obstruction. This is not trivial, as both the mechanism for AF and treatment efficacy appear different for HCM-related AF compared to AF occurring in non-HCM patients [1,4,13]. Our high rates of recurrence are likely related to an underlying substrate that predisposes HCM patients to AF. It has been hypothesized that this substrate represents a specific atrial myopathy in HCM secondary to biophysical abnormalities within ion channels as well as atrial structural remodeling (dilation and fibrosis) and dysfunction that occurs secondary to a genetic predisposition combined with abnormal hemodynamic loading (from outflow obstruction and diastolic dysfunction), as well as comorbidities [4]. This was evident in our cohort, with most patients having outflow obstruction, substantial LA dilation, as well as comorbidities likely impacting both the generation of an initial AF episode and predisposing to risk for recurrence despite AAD utilization. Given the unmet need, there has been a growing number of publications highlighting the importance of this arrhythmia within HCM, often describing the impact of AF on outcomes on a population level, but without granular patient-level details [3,14,15]. To this purpose, we take the opportunity to provide a detailed description of AF episodes, treatment, and recurrence in the immediate time period after first detected AF in a large HCM cohort, allowing a number of novel observations.

The major impetus of this analysis was to describe recurrence rates of AF after both first detected AF as well as after initial treatment interventions, and to this purpose demonstrated high rates of recurrence within all stages of HCM-related AF. We have recently evaluated AF recurrence rates in HCM as part of the large TriNetX database, allowing us to directly compare outcomes in HCM vs. non-HCM patients. However, this analysis was limited, as the nature of the database does not allow identification of discrete AF episodes; rather, only those AF episodes that result in changes in AF treatment interventions [16] can be identified. In contrast, in this well-characterized cohort, we were able to identify all clinically identified AF episodes and not those limited to changes in treatment. After first detected AF, the rate of spontaneous recurrence of AF was about 50% at 1 year. This rate of recurrence is nearly 2.5-fold higher than the 20% of HCM patients requiring changes in AF treatment interventions at 1 year, as reported from TriNetX [16]. The high rate of spontaneous recurrence informs patients and clinicians about the natural history of AF in HCM and informs shared decision-making regarding potential initiation of a rhythm-control strategy after first detected AF, given the relapsing nature of AF for many HCM patients.

It is concerning that we found that recurrence rates were similar in patients treated with AAD (38% at 1-year) compared to those treated solely with rate-control medications. In this regard, about one-third of patients were treated with AAD or catheter ablation after the first detected AF episode, while the remaining two-thirds did not undergo these interventions; rather, the plan was to monitor for clinical AF recurrence prior to intervening. While these findings are subject to the inherent selection bias for more aggressive AF treatment in those patients viewed as higher risk for recurrence, the 38% rate of AF recurrence at 1 year after AAD initiation does raise considerations over the efficacy of these medications in HCM. This is likely based on abnormalities in multiple pathways in the action potential in HCM, which are either not fully targeted by AAD such as sotalol and dofetilide, and/or are avoided in young HCM patients given concern over systemic toxicities such as amiodarone [17,18,19]. Thereby, these data raise priority for well-conducted comparative studies on the efficacy of various AAD specifically in HCM and provide the opportunity for development of HCM-specific AF therapies.

In contrast, we did find a relatively lower rate of AF recurrence after AF ablation, with just 18% having AF recurrence at 1 year and significantly lower than those treated with AAD or followed for spontaneous recurrence. Pulsed field ablation was the most frequently used modality (40% of patients) and was associated with a low recurrence rate, although this difference did not reach statistical significance compared to thermal ablation. This lack of statistical difference likely reflects our smaller sample size. Nonetheless, this trend is numerically consistent with a multicenter HCM cohort that reported significantly lower AF recurrence at 1 year with PFA versus thermal ablation (HR 0.46) [20]. These favorable findings, in combination with other data in both HCM and non-HCM populations demonstrating that ablation is more effective earlier in the natural history of AF, suggest that PFA performed as a primary treatment strategy after first detected AF may result in improved maintenance of sinus rhythm and is deserving of further study [21,22,23,24,25].

Our data also demonstrate the increased morbidity associated with AF related to symptoms. Indeed, 87% of our cohort had EHRA class ≥2, consistent with at least mild symptoms, including ~40% with severe or disabling symptoms (EHRA class 3 or 4), and an additional 23% with moderate symptoms (EHRA class 2b) [10]. In contrast, only a minority (13%) had asymptomatic AF episodes initially detected incidentally or subclinically (on an implanted device), with about one-quarter of these patients ultimately developing symptomatic AF. Thereby, for most HCM patients, AF is highly symptomatic, necessitating rhythm-control interventions to mitigate this additive symptom burden on top of potential symptoms in sinus rhythm from their HCM.

One surprising finding in our cohort was that patients with paroxysmal AF had similar rates of AF recurrence as those with persistent episodes. In non-HCM AF, rates of AF recurrence are lower in paroxysmal episodes driven primarily by PV triggers, and significantly higher in persistent AF, given significantly increased electrical and structural atrial remodeling that drives AF [26,27,28]. As HCM patients are predisposed due to genetic abnormalities and hemodynamic abnormalities resulting in atrial remodeling including dilation, fibrosis, and systolic dysfunction, it is likely that even paroxysmal episodes are driven by abnormal atrial substrate (outside of PV drivers), resulting in these high rates of recurrence regardless of AF type [29,30]. This, in combination with the heterogeneity inherent in HCM, also likely explains the absence of univariate or multivariable predictors for AF recurrence within our cohort. Future studies should include additional variables not available in our cohort, such as cardiac magnetic resonance imaging features (such as atrial or ventricular fibrosis) or biomarkers (such as troponin or BNP levels) to determine if they are better markers to predict recurrent AF events.

Lastly, we identified an important subgroup of 11 patients, representing 7% of the cohort, who had an ischemic stroke before AF was first detected and thereby before the opportunity to initiate anticoagulation for stroke prevention. In these patients, AF was subsequently detected either during the initial stroke work-up (n = 3) or soon after the event (median 0.38 years), making an asymptomatic AF episode a likely mechanism. However, it is possible that substantial atrial dysfunction secondary to an atrial myopathy from HCM was the driver behind these events, predisposing to stroke and subsequent AF and an area deserving of additional study [31]. In contrast, only one thromboembolic event occurred among patients who were initiated on anticoagulation after AF identification, and rates of major bleeding were relatively low (only 2% of patients). These data highlight the clinical importance of early AF detection in HCM and prioritizing mechanisms to allow for early detection, including the role of wearable devices.

Limitations. This is a retrospective analysis from a tertiary referral HCM center, which may introduce selection bias, with the HCM population potentially having more advanced disease. The sample size, particularly within subgroup analyses, was relatively modest, which limited statistical power and may partly explain the lack of significant differences observed between certain groups. To compare different treatment modalities, we extended follow-up in patients undergoing AF ablation by an additional 3 months (to account for a 3-month blanking period in which AF recurrence may not reflect long-term durability of an ablation). Notably, no patient had AF recurrence between 12 and 15 months; thus, this extension does not substantially change our analysis. Additionally, as patients crossed over between treatment strategies over time, the three groups compared in the Kaplan–Meier analysis in Figure 2 were not fully independent, resulting in treatment bias. Treatment strategies were dictated in large part by providers and patients and not randomized, with management decisions inherently influenced by clinician judgment and patient characteristics, limiting comparisons between initial treatment strategies. Lastly, as part of this analysis, we defined the index event as the time that AF was first detected. However, this may not correspond to the biological onset of AF, as a subset of patients are asymptomatic with AF episodes and thereby the true time of initial AF onset is unknown. In this regard, the purpose of this analysis was to describe the clinical history of AF after initial clinical detection. We are unable to analyze the natural history of subclinical AF in HCM from our cohort, as we cannot reliably detect subclinical AF for most of our patients given the absence of implanted monitoring devices.

In conclusion, in this large cohort of patients with HCM and first detected AF, AF was associated with a high burden of recurrence, affecting more than half of patients during follow-up. Symptom burden in AF was particularly high, demonstrating morbidity associated with this arrhythmia. In a subset of patients, presumed cardioembolic stroke occurred prior to AF initial identification, raising priority for early AF detection in HCM. Overall, our findings from a real-world HCM cohort underscore the progressive nature of AF in HCM, the limitations of current rhythm-control strategies, and the central importance of additional dedicated study of AF and treatment interventions specific to HCM.

## Figures and Tables

**Table 1:** Baseline clinical characteristics of 149 patients with HCM and AF.

Characteristic		Patients (n = 149)
Age at first detected AF (median, years)		63 (54, 70)
Age < 50 years		52 (35%)
Age ≥ 70 years		41 (27.5%)
Age at HCM diagnosis (median, years)		57 (47, 65)
Sex (Male %)		94 (63%)
NYHA Class	I	65 (44%)
	II	54 (36%)
	III	30 (20%)
BMI (median)		29 (26, 34)
BMI ≥ 30 kg/m^2^		68 (46%)
Hypertension		82 (55%)
Diabetes		22 (15%)
Chronic kidney disease (GFR < 60 mL/min)		25 (17%)
Obstructive sleep apnea		42 (28%)
CAD		17 (11.4%)
Family history of HCM		33 (22%)
Medications, prior to first detected AF		
Mavacamten		3 (2%)
Betablocker		75 (51%)
Calcium channel blocker		17 (12%)
Disopyramide		4 (2.7%)
Myectomy	Number of patients	32 (21.5%)
	Myectomy prior to first detected AF	20 (62.5%)
Alcohol septal ablation	Number of patients	11 (7.4%)
	Alcohol septal ablation prior to first detected AF	4 (36.4%)
Maximum LV wall thickness, mm		19 ± 3.9
Transverse LA dimension, mm		46 ± 7
LA volume Index mL/m^2^		34.2 (28, 42)
Ejection Fraction (median, %); range		65 (62.5, 70)
EF < 50%		4 (2.7%)
LVOT gradient ≥ 30 mmHg at rest		57 (39%)
LVOT gradient < 30 mmHg at rest and ≥30 mmHg with Valsalva or exercise		22 (15%)
≥30 mmHg on exercise echocardiography		16/22 (73%)
≥30 mmHg with Valsalva		12/22 (55%)
Valsalva only (exercise echocardiography not performed)		6/22 (27%)
Mitral regurgitation ≥ moderate		29 (19%)
Apical HCM		18 (12%)
Apical Aneurysm		6 (4%)
CMR performed		124 (83%)
LGE present		88 (71%)
LGE extent (median, %)		4% (1, 9.5)
LGE ≥ 15% of LV mass		12 (10%)
Time from CMR to first detected AF, months (median) *		0.9 (−4.1, 36.5)
HCM-AF score (mean, SD)		23 (4)
ICD placement		36 (24%)
Risk factors leading to ICD placement	Massive hypertrophy	9 (25%)
	Syncope	10 (27.8%)
	Family history of SCD	8 (22.2%)
	NSVT on Holter	26 (72.2%)
	Apical Aneurysm	6 (16.7%)
	End-stage HCM with EF < 50%	4 (11.1%)
	OOHCA	3 (8.3%)

Values are presented as mean (standard deviation), median (interquartile range), or n (%). * Negative values indicate CMR performed after first detected AF. Abbreviations: AF: atrial fibrillation; HCM: hypertrophic cardiomyopathy; NYHA: New York Heart Association; CAD: coronary artery disease; BMI: body mass index; CMR: cardiovascular magnetic resonance; EF: ejection fraction; GFR: glomerular filtration rate; ICD: implantable cardioverter-defibrillator; LA: left atrial; LGE: late gadolinium enhancement; LV: left ventricular; LVOT: left ventricular outflow tract; NSVT: nonsustained ventricular tachycardia; SCD: sudden cardiac death; OOHCA: out-of-hospital cardiac arrest.

**Table 2:** Univariate and multivariable Cox proportional hazards analysis for predictors of atrial fibrillation recurrence during follow-up for patients not initially treated with a rhythm-control strategy.

Variable	HR (95% CI)	*p*-Value
Age	1.017 (0.995–1.040)	0.128
BMI ≥ 30	0.721 (0.426–1.221)	0.225
HCM-AF score ≥ 22	1.381 (0.788–2.420)	0.260
LA dimension	1.014 (0.977–1.052)	0.468
LVOT gradient ≥ 30 mmHg	1.305 (0.762–2.233)	0.332
Electrical cardioversion	1.086 (0.608–1.940)	0.781
Persistent AF at presentation	1.086 (0.608–1.940)	0.781
Obstructive sleep apnea	1.403 (0.792–2.486)	0.247
Maximal LV wall thickness	0.985 (0.922–1.053)	0.661
**Multivariable analysis (n = 98; 59 events)**	**Adjusted HR (95% CI)**	***p*-value**
Age (per year)	1.016 (0.993–1.041)	0.181
LA transverse dimension (per mm)	1.013 (0.974–1.054)	0.514
Resting LVOT obstruction (≥30 mmHg)	1.184 (0.640–2.188)	0.590
Male sex	1.043 (0.578–1.884)	0.888
BMI (per kg/m^2^)	0.980 (0.934–1.028)	0.411
NYHA class ≥ II	1.146 (0.593–2.214)	0.686

HR = hazard ratio; CI = confidence interval; LVOT = left ventricular outflow tract; BMI = body mass index. The multivariable model includes all six covariates simultaneously; time to recurrence was analyzed in days, and one patient with recurrence at time zero was assigned 0.5 days. Global test of the proportional hazards assumption *p* = 0.44 (BMI, *p* = 0.059).

**Table 3:** Atrial fibrillation recurrence and rhythm-control strategies.

Characteristic			Patients (n = 149)
Age at First Detected Atrial Fibrillation (median, years)			63 (54, 70)
Class NYHA just before Atrial Fibrillation		I	65 (44%)
		II	54 (36%)
		III	30 (20%)
European Heart Rhythm Association		1 No symptoms	19 (13%)
		2a Mild symptoms	40 (27%)
		2b Moderate symptoms	34 (23%)
		3 Severe symptoms	25 (17%)
		4 Disabling symptoms	31 (20%)
Number of Atrial Fibrillation Episodes		1	69 (46%)
		2	43 (29%)
		3	19 (13%)
		4	10 (7%)
		5	2 (1%)
		6	6 (4%)
First Detected Atrial Fibrillation Status		Paroxysmal	98 (66%)
		Persistent	51 (34%)
First Detected Atrial Fibrillation Episode Treatment		Self-converted	87 (58%)
		Converted with anti-arrhythmic drug	12 (8%)
		Converted after electrical cardioversion	44 (30%)
		Sent directly to ablation	3 (2%)
		Permanent atrial fibrillation (rate control)	3 (2%)
Started on anti-arrhythmic drug after first detected AF		None	117 (78.5%)
		Amiodarone	20 (13%)
		Sotalol	7 (4.7%)
		Dofetilide	1 (0.7%)
		Disopyramide	4 (2.7%)
Recurrent Atrial Fibrillation Episodes			80 (54%)
Days Between First Detected and Recurrent Atrial Fibrillation (Median, Days)			171 (53, 278)
Patients submitted to cardioversion after first episode of AF who had recurrence		Total patients who were cardioverted during the 1st episode	44 (29.5%)
		Recurrence after cardioversion	24 (54%)
		Time of recurrence (days) among patients who were cardioverted after 1st episode	Median: 145 (22, 333)
Medications used for Atrial Fibrillation treatment during clinical course		Betablocker	106 (71.1%)
		CCB	30 (20.1%)
		Amiodarone	39 (26.1%)
		Sotalol	15 (10%)
		Dofetilide	9 (6%)
		Dronedarone	2 (1.3%)
		Disopyramide	6 (4%)
		Warfarin	1 (0.7%)
		DOAC	137 (91.9%)
		Not on anticoagulation	11 (7.4%)
Atrial Fibrillation Ablation	Number of Ablations	Total	49 (33%)
		1 Ablation	42 (86%)
		2 Ablations	7 (14%)
	Time of follow-up since AF ablation (median, days)		525 (327, 721)
	Recurrent AF after first ablation	Total	13 (27%)
		Time of AF recurrence after AF ablation (days)	Median 262 (122, 402)
Maze procedure	Number of Maze procedures	Total	12
		AF recurrence after Maze	1 (8%)

Values are presented as median (interquartile range) or n (%). Abbreviations: AF = atrial fibrillation; DOAC = direct oral anticoagulant; CCB = calcium channel blocker; NYHA = New York Heart Association; Maze = surgical Maze procedure.

**Table 4:** Outcomes in HCM patients with AF.

Outcome			Patients (n = 149)
Age at last follow-up			Median 67.8 [IQR 61.2–74.7]
Follow-up from first detected AF (years)			Median 1.9 [IQR 1.2–2.6]
NYHA class at last follow-up			Class I: 90 (60.4%) Class II: 45 (30.2%) Class III: 14 (9.4%)
Sinus rhythm at last follow-up			133 (89%)
Died			7 (4.7%)
Age of death (years)			68 (60,77)
Cause of death	Non-HCM death		4 (57%)
	HCM death		3 (43%)
		Complete heart block	1
		Post-op myectomy	1
		Intracranial hemorrhage	1

Abbreviations: HCM = hypertrophic cardiomyopathy; IQR = interquartile range; NYHA = New York Heart Association.

## Data Availability

The data presented in this study are available on reasonable request from the corresponding author. The data are not publicly available due to privacy and ethical restrictions.

## Supplementary Materials

The following supporting information can be downloaded at: https://www.mdpi.com/article/10.3390/jcdd13100517/s1, Table S1: Baseline characteristics of patients treated with antiarrhythmic drugs (n = 32) or catheter ablation (n = 49).
