# Supplementary material for: Recurrence Rates and Outcomes After First Detected Atrial Fibrillation Episode in a Contemporary Hypertrophic Cardiomyopathy Center
Source: J Cardiovasc Dev Dis. 2026 Oct 14;13(10):517. doi: 10.3390/jcdd13100517 (PMC13705043; doi:10.3390/jcdd13100517)
Supplement: Supplementary file 1 [file jcdd-13-00517-s001.zip › jcdd-4550244-supplementary.pdf]

Supplementary Table S1. Baseline characteristics of patients treated with antiarrhythmic drugs (n = 32) or catheter ablation (n = 49).

**Characteristic**	**AAD (n = 32)**	**Catheter ablation (n = 49)**
Age at first detected AF, years	68 (57, 73)	63 (57, 68)
Male sex	13 (41%)	37 (76%)
BMI, kg/m²	28 (26, 34)	31 (26, 33)
BMI ≥30 kg/m²	14 (44%)	25 (51%)
Hypertension	18 (56%)	27 (55%)
Obstructive sleep apnea	11 (34%)	22 (45%)
Diabetes	4 (12%)	8 (16%)
NYHA class ≥II prior to first detected AF	23 (72%)	23 (47%)
Maximum LV wall thickness, mm	19.7 ± 4.2	18.9 ± 2.7
Transverse LA dimension, mm	46 ± 8	47 ± 8
Resting LVOT gradient ≥30 mmHg	18 (56%)	13 (27%)
HCM-AF score	24 ± 4	24 ± 4
Persistent first detected AF	13 (41%)	24 (49%)
LGE present	16/22 (73%)	34/44 (77%)
AF recurrence after treatment	14 (44%)	13 (27%)

Values are presented as median (interquartile range), mean ± standard deviation, or n (%). Eight patients underwent catheter ablation after AAD failure and are included in both columns. LGE is expressed among patients who underwent CMR. AAD = antiarrhythmic drug; AF = atrial fibrillation; BMI = body mass index; CMR = cardiovascular magnetic resonance; HCM = hypertrophic cardiomyopathy; LA = left atrial; LGE = late gadolinium enhancement; LV = left ventricular; LVOT = left ventricular outflow tract; NYHA = New York Heart Association.
